# A computational model for microbial colonization of an antifouling surface

**DOI:** 10.3389/fmicb.2022.920014

**Published:** 2022-09-27

**Authors:** Patrick Sinclair, Jennifer Longyear, Kevin Reynolds, Alistair A. Finnie, Chris A. Brackley, Martín Carballo-Pacheco, Rosalind J. Allen

**Affiliations:** ^1^School of Physics and Astronomy, University of Edinburgh, Edinburgh, United Kingdom; ^2^Marine, Protective and Yacht Coatings, International Paint Ltd, AkzoNobel, Gateshead, United Kingdom; ^3^Theoretical Microbial Ecology, Institute of Microbiology, Faculty of Biological Sciences, Friedrich-Schiller University Jena, Jena, Germany

**Keywords:** computational modeling, marine biofouling, antifouling paint, stochastic model, biofilm establishment

## Abstract

Biofouling of marine surfaces such as ship hulls is a major industrial problem. Antifouling (AF) paints delay the onset of biofouling by releasing biocidal chemicals. We present a computational model for microbial colonization of a biocide-releasing AF surface. Our model accounts for random arrival from the ocean of microorganisms with different biocide resistance levels, biocide-dependent proliferation or killing, and a transition to a biofilm state. Our computer simulations support a picture in which biocide-resistant microorganisms initially form a loosely attached layer that eventually transitions to a growing biofilm. Once the growing biofilm is established, immigrating microorganisms are shielded from the biocide, allowing more biocide-susceptible strains to proliferate. In our model, colonization of the AF surface is highly stochastic. The waiting time before the biofilm establishes is exponentially distributed, suggesting a Poisson process. The waiting time depends exponentially on both the concentration of biocide at the surface and the rate of arrival of resistant microorganisms from the ocean. Taken together our results suggest that biofouling of AF surfaces may be intrinsically stochastic and hence unpredictable, but immigration of more biocide-resistant species, as well as the biological transition to biofilm physiology, may be important factors controlling the time to biofilm establishment.

## 1. Introduction

Marine biofouling is a pervasive problem in the shipping industry. Biofilm formation on ship hulls increases hydrodynamic drag, resulting in higher fuel consumption which leads to higher economic and environmental costs (Bott, 2011; Schultz et al., 2011). This is a major issue, since around 90% of the world's trade is transported *via* the shipping industry (Banerjee, 2017), accounting for 2.2% of global greenhouse gas emissions (Yeeles, 2018; IMO, 2020).

Marine biofouling of a newly immersed surface is a dynamic process that is influenced by factors such as availability of colonizers, local environmental conditions and species interactions. Several stages are commonly observed during the formation of biofouling (Callow and Callow, 2011). Within a few seconds of a surface being submerged in the marine environment, it becomes covered by a conditioning layer of dissolved proteins and other organic detritus. The surface can then become colonized by microbes in a matter of hours, resulting in the formation of a biofilm. Finally, in the macrofouling stage, larger marine invertebrates such as barnacles or mussels attach (Callow and Callow, 2002). Progression from one stage to the next is not causal, but interactions between fouling species can influence the patterns of colonization and biofouling accumulations (Callow and Callow, 2011). In particular, the microbial biofilm facilitates the attachment of the larger fauna (Dobretsov and Qian, 2006; Qian et al., 2007), and there is evidence that prospective macrofoulers can differentiate between biofilms with different microbial species composition (Patel et al., 2003; Lau et al., 2005). While macrofouling is the major contributor to drag and ship hull degradation (Leer-Andersen and Larsson, 2003), the microbial biofilm itself can also contribute significantly to the increased drag on the ship (Lewthwaite et al., 1985; Barton et al., 2007; Andrewartha et al., 2010).

Many researchers aim to develop novel alternative technologies to limit the growth of biofilms and the subsequent attachment of macrofoulers on the outer hulls of ships and boats. For example ultrasound (Legg et al., 2015), UVC-emitting surfaces (Salters and Piola, 2017), and regular proactive surface cleaning (“grooming”) (Swain et al., 2022) are often perceived as being relatively environmentally-benign solutions. However, in practice, vessels are generally coated with specialist paints and while biocide-free “fouling-release” paints are available, and are successfully used on many vessels, they reportedly account for only 5–10% of sales by volume for the commercial shipping sector (Bressy and Lejars, 2014). For now, biocidal antifouling (AF) paints, which contain and release biocide, are still very widely used.

It has been estimated that AF coatings reduce the fuel costs of the shipping industry by *$*60 billion each year, as well as lowering yearly emissions of carbon dioxide and sulphur dioxide by 384 million and 3.6 million tonnes, respectively (figures estimated in 2010, Salta et al., 2010). The most commonly used types of biocidal AF paint—self-polishing and ablative coatings—are designed such that the matrix of the paint solubilizes slowly in seawater, ensuring a relatively controlled and constant biocide release rate (Thomas et al., 1999; Chambers et al., 2006; Ma et al., 2017). Modern biocidal AF paints often use an inorganic copper compound, particularly cuprous oxide, in conjunction with an organic or metal-organic compound such as 4,5-Dichloro-2-n-octyl-4-isothiazolin-3-one (DCOIT), copper pyrithione or zineb as co-biocides to provide broad spectrum protection against the wide range of marine fouling organisms that may be encountered (Finnie and Williams, 2010). The paint product used on any particular vessel is generally selected on the basis of the customer's expectations for cost vs. performance. Furthermore, in many countries, including EU countries, UK, USA, Canada, China, Australia and New Zealand, the use of biocidal AF paints is increasingly tightly controlled by regulation in response to environmental concerns associated with the release of biocide into marine waters (Pereira and Ankjaergaard, 2009).

While most commercial antifoulings are effective at preventing the growth of marine fouling on most vessels over the required service period, which may be up to 7.5 years, no single product is effective at preventing all fouling on all vessels. The onset of fouling can be hard to predict and among the primary variables are likely to be vessel operational profile and environmental factors (Kidd et al., 2016). Commonly, some level of microbial fouling over the 5–7 year docking cycle is observed on ship hulls protected by biocidal paints. However, as biofilm fouling also causes increased frictional drag, paints which minimize slime formation are advantageous. Understanding how AF paints affect microbial biofilms is therefore essential so as to design and utilize them with maximal effectiveness and minimal environmental impact.

Here, we present a computational model for the colonization of an AF surface by a multispecies microbial community. Our model predicts biofilm formation dynamics and provides insight into the microbial diversity of the biofilm. Our simulations suggest that biofilm formation on the AF surface can be stochastic, with an exponential distribution of waiting times before biofilm establishment. In our model, the average time before significant biofilm accrues on a surface depends exponentially on both the concentration of biocide and the rate of arrival of resistant organisms from the ocean. Taken together our model puts forward a picture in which biocide-resistant organisms immigrate stochastically from the ocean, and eventually trigger biofilm formation in a process that can itself be stochastic. In our model, once biofilm growth is established, the outer part of the biofilm is shielded from the biocide and can support the growth of more biocide-susceptible organisms. Our work should provoke debate about the mechanisms controlling biofouling of AF surfaces under different parameter regimes and the extent to which the biofouling process may be inherently stochastic and unpredictable.

## 2. Methods

### 2.1. A computational model for biofilm growth on an AF surface

**Figure 1:**
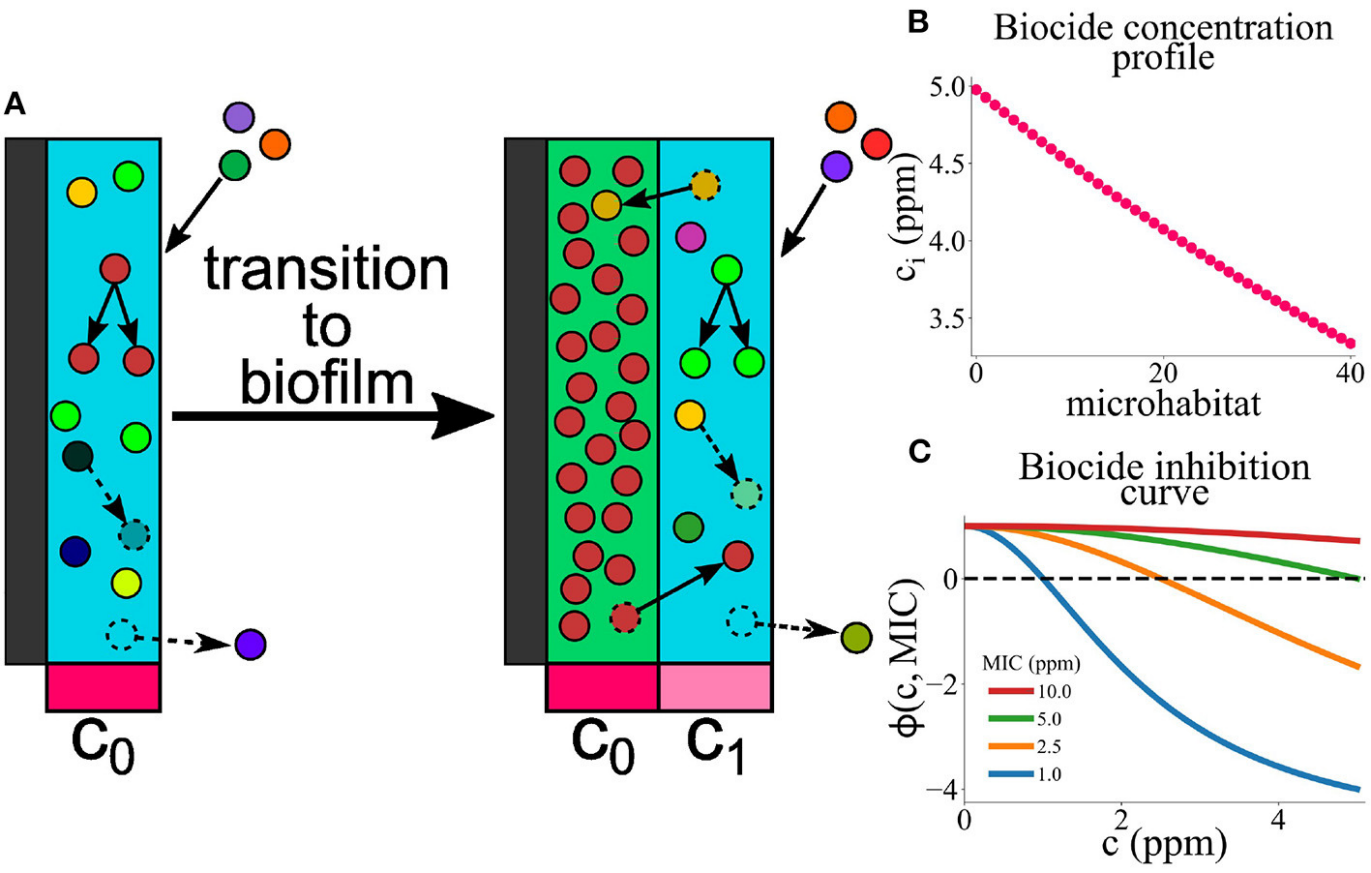
**(A)** A model for microbial colonization of an AF surface. Microbes immigrate from the well-mixed marine environment into the edge microhabitat. They can replicate, die, migrate between adjacent microhabitats, or detach from the edge microhabitat. Once the population of the edge microhabitat reaches a threshold size, a new edge microhabitat is added. This creates an expanding series of microhabitats, representing the growth of a marine biofilm. Each microhabitat *i* contains a concentration of biocide, *c*_*i*_, which decreases exponentially with distance from the surface. **(B)** Biocide concentration *c*_*i*_ as a function of microhabitat index *i*. The biocide concentration has a maximum value *c*_max_ (here 5 ppm), and decreases exponentially in successive microhabitats; note that we only simulate up to a system size of 40 microhabitats. **(C)** Biocide inhibition curves (pharmacodynamic function ϕ) as a function of biocide concentration *c* for microbes with MIC values between 1 and 10 ppm. Positive values of ϕ indicate microbial growth; negative values of ϕ indicate microbial death. The dashed black line represents the boundary between microbial growth and death. Microbes with lower MIC values are more susceptible to the biocide and therefore die at lower biocide concentrations.

We present a model for biofilm deposition and growth on a marine AF surface (Figure 1). To capture the key aspects—the spatial gradient of biocide as it diffuses away from the surface and the multispecies nature of the biofilm—in a computationally efficient manner, we use a coarse-grained “microhabitat” modeling approach (Greulich et al., 2012; Allen and Waclaw, 2019; Sinclair et al., 2019) (also widely known as a “deme” modeling approach). The biofilm is modeled as a series of slices, here called microhabitats, labeled with index *i* that runs from *i* = 0 to *L*. The first microhabitat (*i* = 0) is immediately adjacent to the AF surface and subsequent microhabitats extend into the marine environment (Figure 1). Each microhabitat contains a different concentration of biocide, representing the concentration gradient that results from diffusion of biocide from the surface (Figure 1B).

In the model, we track the population density of microbes within each microhabitat. Microbes are introduced to the system *via* immigration from the marine environment. Rather than assigning a taxon to each microbe, we categorize microbes according to their level of resistance to the biocide, defined by a minimal inhibitory concentration (MIC) value. Therefore in some sense our model can be viewed as an ecotype model, where “ecotype” here refers to the level of biocide resistance. The biocide resistance of immigrant microbes is chosen from a distribution, such that highly biocide-resistant species are rare.

Initially, microbes immigrate into the first microhabitat (adjacent to the surface) and form a loosely-attached layer, proliferating or dying according to their level of resistance to the biocide. If the local biocide concentration exceeds the MIC for a particular microbe, that microbe will tend to die, whereas if the biocide concentration is less than the MIC value, it will proliferate (Figure 1C). When the population density in the first (surface) microhabitat reaches a threshold size, the biofilm expands into the next microhabitat; further immigrants then attach to the new outer microhabitat. This process continues, with new microhabitats being added as the population in the outermost one reaches a threshold, such that the biofilm expands outwards. Therefore the number *L* of microhabitats increases as the simulation progresses. Microbes within the biofilm can replicate, die, migrate between adjacent microhabitats or, in the outermost microhabitat only, detach from the biofilm.

We simulate this model using a stochastic agent-based approach which tracks the number of microbes of each biocide-resistance level in each microhabitat. The key model parameters are: the maximal biocide concentration *c*_max_ at the surface-seawater interface, the steepness *α* of the biocide gradient, the parameters *μ* and *σ* of the log-normal MIC distribution of the immigrating microbes (which control the mean MIC value for immigrants and the percentage of immigrants with MIC above *c*_max_), the microbial immigration rate *r*_imm_, the maximum rate of microbial growth *r*_max_, which also controls the maximal rate of biocide killing, the carrying capacity *K* of a microhabitat (which depends on the microhabitat thickness *δz* and lateral area *δa*), the population size N^*^ at which a microhabitat transitions to the biofilm state (which also depends on *δz* and *δa*), the detachment rate *r*_det_, the rate *r*_mig_ of migration of microbes within the biofilm, and the biocide-independent microbial mortality *d*_uniform_.

We now describe in more detail the components of our model and the parameter values.

#### 2.1.1. Biocide gradient

We assume that the concentration of biocide decreases exponentially with distance away from the AF surface. This is consistent with a scenario in which biocide diffuses from the surface and is degraded at a uniform rate (Supplementary material). Therefore, in our model, the concentration *c*_*i*_ of biocide in the *i*-th microhabitat is given by


$$\begin{array}{l} c_{i}=c_{\max}e^{-\alpha \left(\frac{i+1}{2}\right)\delta z}, \end{array}$$

where $\left(\frac{i+1}{2}\right)\delta z$ represents the midpoint of the *i*-th microhabitat.

#### 2.1.2. Biofilm initiation and expansion

In our model, microhabitats can be in one of two possible states: A “pre-biofilm”, in which microbes are loosely attached, with a low population density, and B “biofilm”, in which microbes are more strongly attached (Sinclair et al., 2022). A microhabitat transitions from state A to state B when its microbial population reaches a critical value N^*^. When this happens, a new microhabitat (in state A) is created. This model mimics a quorum-sensing-mediated transition from planktonic to biofilm physiology (see Section 4, Moore-Ott et al., 2022; Sinclair et al., 2022). Our simulations are initialized with one microhabitat (*i* = 0, *L* = 0) in state A, adjacent to the surface. Once the population in this first microhabitat reaches N^*^, a second microhabitat is created, adjacent to the first one. Thus the growing biofilm is modeled as a series of connected microhabitats extending away from the surface into the ocean.

#### 2.1.3. Microbial immigration

New microbes are introduced into the outermost microhabitat at rate *r*_imm_. To mimic the microbial diversity of the marine environment, we classify microbes according to their degree of biocide resistance. Thus, each immigrating microbe is assigned a numerical value denoting its minimum inhibitory concentration (MIC) of biocide (see below). This MIC value serves as a form of ecotype identifier and is inherited upon proliferation.

To our knowledge, the distribution of biocide MIC values for marine microbes has not yet been characterized. MIC values for bacteria more generally have been found to be log-normally distributed (Turnidge et al., 2006), therefore we assume that the biocide MIC values for microbes immigrating from the ocean follow a log-normal distribution :


$$\begin{array}{l} P\left(x\right)=\frac{1}{x\sigma \sqrt{2\pi }}\exp\left(-\frac{{\left(\ln\;x-\mu \right)}^{2}}{2\sigma^{2}}\right), \end{array}$$

where *P*(*x*) is the probability of obtaining MIC value *x* and the parameters *μ* and *σ* control the mean and width of the distribution (specifically the mean MIC value is given by ${\text{MIC}}_{\text{ave}}=\exp\left(\mu +\sigma^{2}/2\right)$ and the probability of obtaining an MIC value greater than a threshold MIC_*t*_ is $\left[1-\text{erf}\left(\left(\ln{\text{MIC}}_{t}-\mu \right)/\left(\sigma \sqrt{2}\right)\right)\right]/2$). We used an in-house computational code to set the values of *μ* and *σ* to achieve a chosen mean MIC and a chosen percentage of immigrating microbes with MIC higher than the surface biocide concentration *c*_max_. We then sampled MIC values from the log-normal distribution using standard methods (Press et al., 2007).

#### 2.1.4. Detachment and migration

Microbes are removed from the outermost microhabitat (which is in the loosely attached state (i); see above) at rate *r*_det_. Microbes also move between adjacent microhabitats at rate *r*_mig_.

#### 2.1.5. Microbial proliferation and death

Within a given microhabitat, microbes proliferate if the local biocide concentration is lower than their MIC, and die if the biocide concentration exceeds their MIC. Following previous work (Regoes et al., 2004; Greulich et al., 2012; Sinclair et al., 2019), we model the rate of proliferation/biocide killing using the following pharmacodynamic function (Regoes et al., 2004):


$$\begin{array}{l} \phi \left(c,\text{MIC}\right)=r_{\text{max}}\left(1-\frac{6{\left(c/\text{MIC}\right)}^{2}}{5+{\left(c/\text{MIC}\right)}^{2}}\right) \end{array}$$

This function is positive if the concentration *c* is less than the MIC, and negative if *c* > MIC. It is a specific case of the general function proposed by Regoes et al. (2004), which we have used in previous work (Greulich et al., 2012; Sinclair et al., 2019); similar functions would produce equivalent results. Since microbial mortality in the ocean is high even in the absence of biocide (Servais et al., 1985; Pace, 1988; Menon et al., 2003), we also include a uniform turnover rate *d*_uniform_ for all microbes, irrespective of the biocide concentration. Finally, we account for the finite supply of nutrient and space within a microhabitat by including a logistic growth term 1 − *N*/*K*, with carrying capacity *K*, such that growth slows as the population size *N* in a given microhabitat approaches the carrying capacity (Tsoularis and Wallace, 2002).

In summary, in a microhabitat with biocide concentration *c* and microbial population *N*, microbes with a given MIC behave as follows. If *c* < MIC, they proliferate at rate $\phi \left(c,\text{MIC}\right)\left(1-\frac{N}{K}\right)$ while simultaneously dying at rate *d*_uniform_. If *c* > MIC, these microbes do not proliferate, but instead they die at rate |*ϕ*(*c*, MIC)| + *d*_uniform_. Daughter microbes retain the same biocide resistance level as the mother; i.e., mutations are not included in the model.

#### 2.1.6. Simulation algorithm

**Figure 2:**
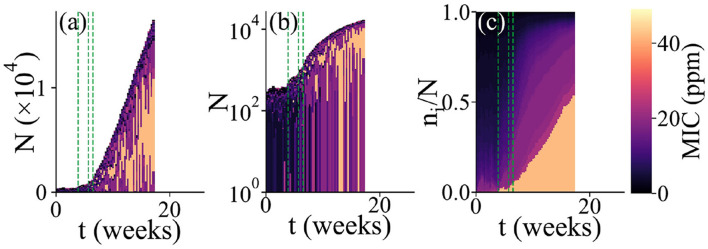
Simulation of microbial colonization of an AF surface. An example of a simulation run in which a biofilm is established. The population composition vs. time *t* is represented in 3 different ways. In all cases, the colors represent the resistance levels (MIC, in ppm) of microbes within the population (see color scale). For each time point, a vertical bar shows the state of the population; these bars are stacked adjacent to each other to show dynamical changes. This run stopped when the biofilm reached the thickness limit of 40 microhabitats. The green dashed lines represent times at which new microhabitats were added to the system. For clarity, only the first 3 such events are shown. **(a)** Total population size and composition. Here, the bar height represents the total population size. The colors show the resistance levels within the population; here, individual bars for each microhabitat are stacked such that the lower part of each bar represents the region of the biofilm close to the surface while the upper part represents the region further from the surface. **(b)** Same plot as in **(a)**, but with a log scale on the vertical axis. **(c)** Relative population composition. Here, the colors represent the resistance levels present in the population, as fractions of the total population.

**Figure 3:**
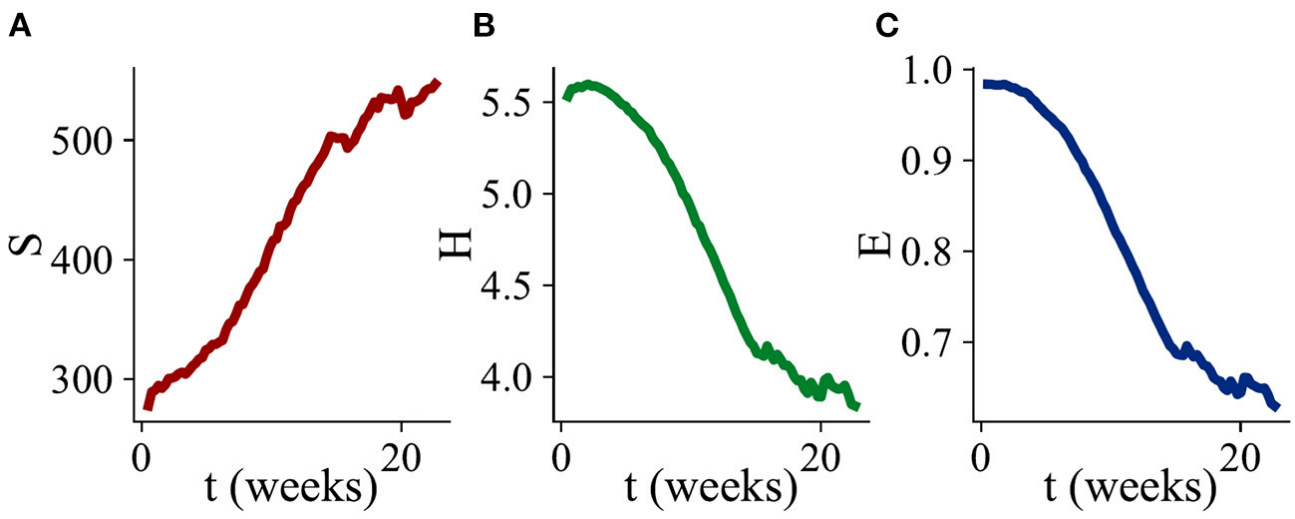
Changes in alpha diversity during biofilm development. Three diversity indices are computed, defining a “species” as a microbial type with a distinct MIC value. Values of the diversity indices are averaged over all of the simulation runs which exhibited biofilm growth. **(A)** Average number of species *S* as a function of time. **(B)** Average Shannon index *H* as a function of time. **(C)** Average Shannon equitability *E* as a function of time. While *S* increases with time, *H* and *E* both decrease.

**Figure 4:**
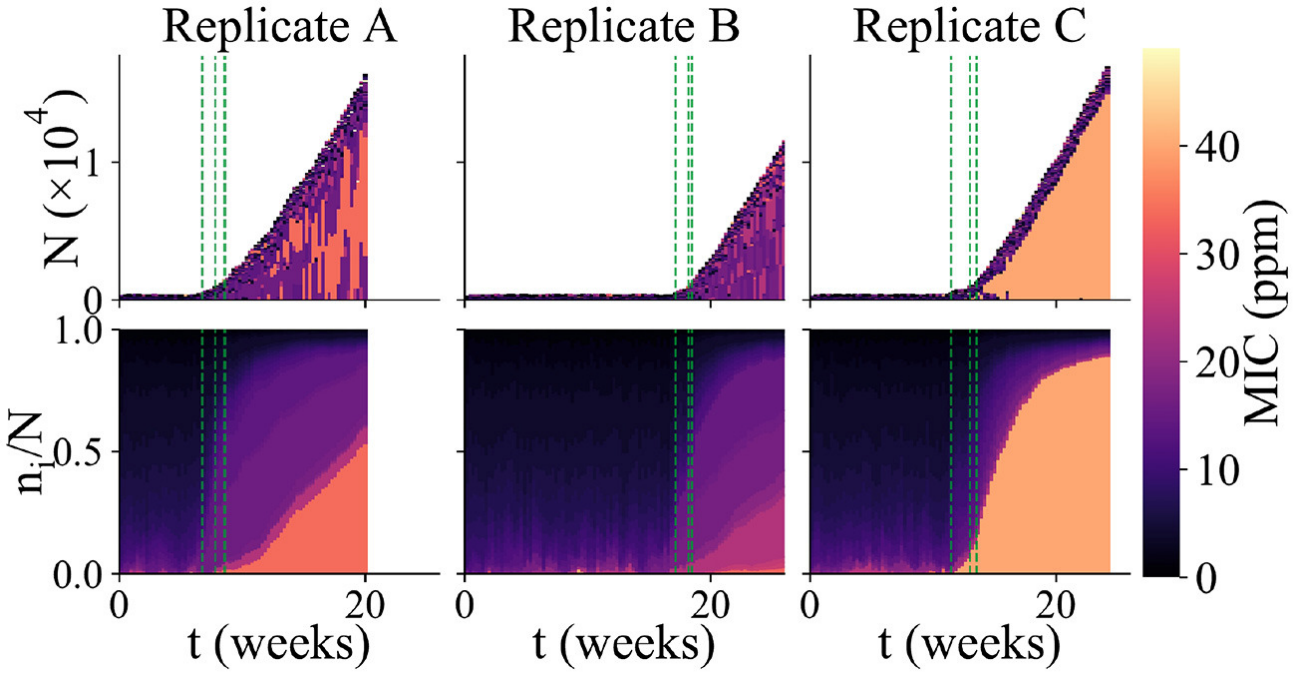
Variability among replicate simulation runs. Community composition of 3 replicate simulations runs in which biofilm formed (each column shows an independent simulation run). The upper panels show total community size and composition (as in Figure 2a), while the lower panels show the relative abundance of microbes with different MIC values (as in Figure 2c). The color scale indicates MIC value. As in Figure 2, the green dashed lines indicate the times at which new microhabitats are added (for the first 3 microhabitats only). Replicate A shows an example of a run which reached the “thickness limit” and stopped early.

**Figure 5:**
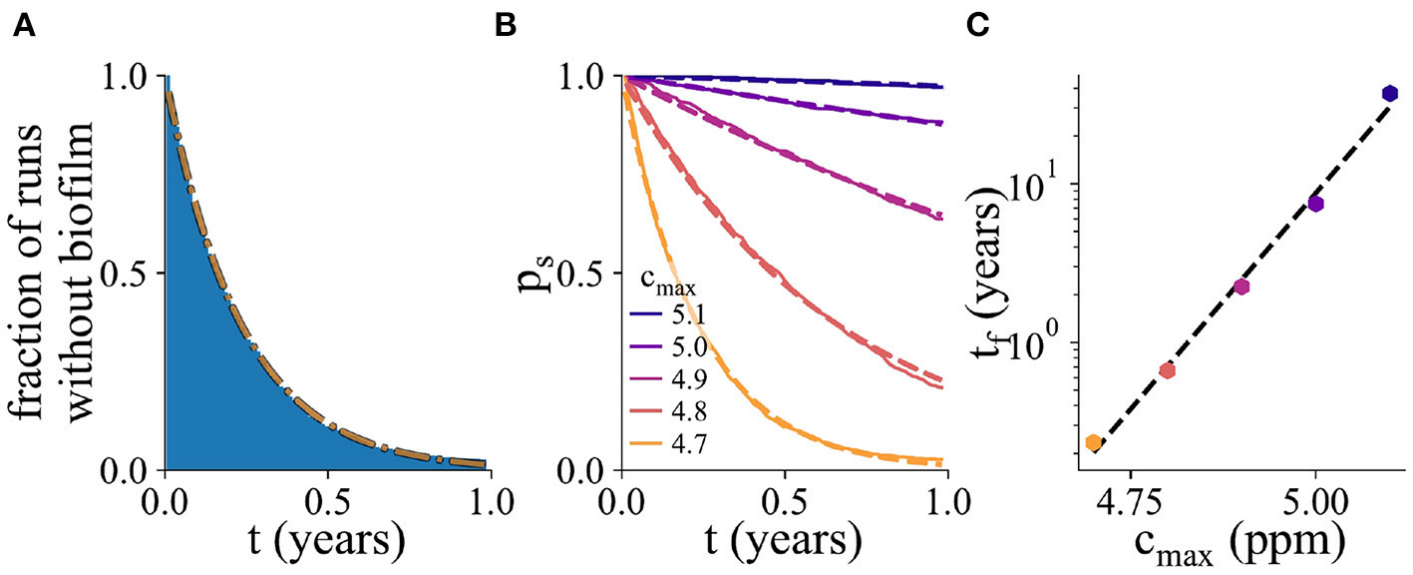
Probability of biofilm establishment. **(A)** Normalized histogram (blue) of the number of replicate simulation runs in which biofilm has not yet formed by time *t*, for 2,000 replicate simulations, for *c*_max_ = 4.7ppm. The fitted exponential probability distribution, *p*_*s*_(*t*), is shown in orange. **(B)** The probability distribution *p*_*s*_(*t*), for a range of values of *c*_max_. Here, the percentage of resistant microbes is set to 14% for the *c*_max_ value of 5 ppm. **(C)** The mean biofilm establishment time, *t*_*f*_, as a function of *c*_max_. The mean biofilm establishment time increases exponentially with *c*_max_.

**Figure 6:**
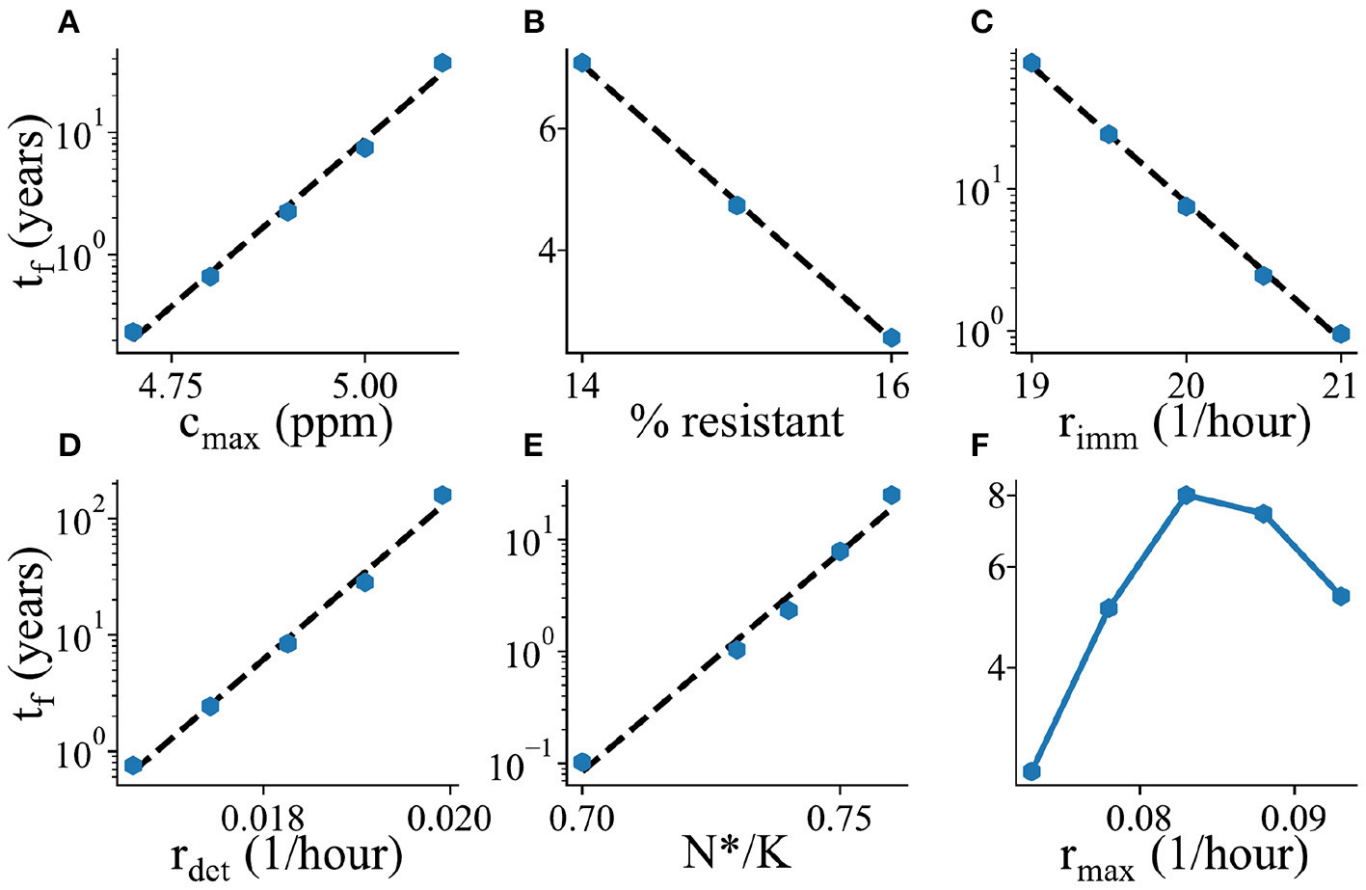
Parameter dependence of mean biofilm establishment time. The mean biofilm establishment time *t*_*f*_ is plotted as a function of various model parameters. **(A)** Maximal biocide concentration *c*_max_, **(B)** Percentage of biocide resistant microbes in the ocean, **(C)** Immigration rate *r*_imm_, **(D)** Detachment rate *r*_det_, **(E)** Biofilm transition threshold N^*^/*K*, **(F)** Maximal growth/biocide killing rate *r*_max_. All plots are shown with a log-scale on the *y*-axis.

The model was simulated using a tau-leaping algorithm (Gillespie, 2001), which takes account of the stochasticity of individual immigration, migration, birth, death and detachment events. The algorithm is modified compared to the standard tau-leaping algorithm to avoid negative population sizes (Cao et al., 2005); see also Supplementary material. For the data shown in Figures 2–4, the simulations were continued until either 6 months of simulated time had elapsed or the biofilm had grown to a thickness of 40 microhabitats. For the biofilm establishment time data shown in Figures 5, 6, the simulated time was increased to 1 year.

#### 2.1.7. Model parameters

The parameter values used in our simulations are listed, together with their sources, in Table 1. For some parameters, further explanation is given in Supplementary material. Importantly, our parameter set is in the “stochastic biofilm initiation regime” identified in previous work (Sinclair et al., 2022). This means that the predicted population size in the first microhabitat is below the biofilm threshold N^*^, even for a microbe that is fully biocide-resistant. Therefore we expect to see initial loose colonization of the first microhabitat, with a population size below the threshold N^*^, before a stochastic fluctuation in the population size pushes the system over the threshold, triggering biofilm formation (Sinclair et al., 2022).

**Table 1:** Parameters used in our computational model.

**Parameter**	**Definition**	**Value**	**Source/Rationalization**
*δz*	Microhabitat thickness	1 *μ*m	Approx. width of one microbial layer
*δa*	Microhabitat lateral area	0.5 mm × 0.5 mm	Implies assumed lateral diffusion area for QS signals (van Gestel et al., 2021); see Section 4.5
*r*_max_	Max. growth rate, controls biocide kill rate	0.083 h^−1^ (varied in Figure 6)	Growth rates observed for marine bacteria (Middelboe, 2000; Ploug and Grossart, 2000; Grossart et al., 2003)
*d*_uniform_	Uniform death rate	0.018 h^−1^	Ocean mortality (Servais et al., 1985; Pace, 1988; Menon et al., 2003)
*K*	Carrying capacity of microhabitat	550 microbes (2.2 × 10^6^mm^−3^)	Marine biofilm density on fouling-release coatings (Dobretsov and Thomason, 2011)
*N* ^*^	Population threshold for biofilm transition	0.75 × *K*	Adjusted to biofilm growth rate (Dobretsov and Thomason, 2011); see Supplementary material
MIC_ave_	Average biocide MIC	3.179 ppm	Adjusted to fix overall killing rate; see Supplementary material
*μ*	MIC distibution scale parameter: mean of the normally distributed natural logarithm of MIC distribution,	2.48 (Figures 2–4); varied in Figure 6	Set to achieve desired MIC_ave_ and pc_res_
*σ*	MIC distribution shape parameter: standard deviation of the normally distributed natural logarithm of MIC distribution	0.71 (Figures 2–4); varied in Figure 6	Set to achieve desired MIC_ave_ and pc_res_
pc_res_	% of immigrants with MIC>*c*_max_	16% (Figures 2–4; varied in Figure 6	No data available to our knowledge
*c*_max_	Maximal biocide concentration at seawater interface	5 ppm (varied in Figures 5, 6)	Assume to be controlled by biocide solubility in seawater, e.g., 4.7 ppm for Kalthon930 (O'Neil, 2013)
α	Biocide gradient parameter	0.01 *μ*m^−1^	Consistent with diffusion/degradation; see Supplementary material
*r*_imm_	Immigration rate	20 h^−1^ (varied in Figure 6)	Scaling of values reported by (Fletcher and Loeb, 1979); see Supplementary material
*r*_mig_	Migration rate	0.1 h^−1^	Scaling of values for *Pseudomonas* biofilms (Rice et al., 2003); further decreased by factor of 10 for computational convenience
*r*_det_	Detachment rate	0.22 × *r*_max_ (varied in Figure 6)	Adjusted to biofilm growth rate (Dobretsov and Thomason, 2011); see Supplementary material
*t*_max_	Maximum simulation time	6 months (Figures 2–4); 1 year (Figures 5, 6)	Computational feasibility
*L*_max_	Maximum biofilm thickness	40 microhabitats	Computational feasibility

## 3. Results

### 3.1. Microbial colonization of an AF surface

Figure 2 shows the results of a typical simulation run in which the AF surface becomes colonized. In Figure 2a, the dynamics of biofilm development are represented as a series of vertical bars, corresponding to the biofilm population at increasing times. The height of each bar corresponds to the total biofilm population size, illustrating the overall growth dynamics of the biofilm. Within each bar, the colors show the composition of the population in terms of biocide resistance level, from purple (low MIC; susceptible) to orange (high MIC; resistant). To account for the spatial structure of the biofilm, each vertical bar consists of a stack of smaller bars, each corresponding to one microhabitat. Thus, the lower part of each bar represents the region of the biofilm close to the surface while the upper part represents the region further from the surface. Figure 2b shows the same information as Figure 2a but with a log scale on the vertical axis, allowing the early-time dynamics to be more clearly seen. Figure 2c focuses on changes in the microbial community composition as the biofilm develops. Here, the vertical height of the bars is scaled by the population size, and within each bar the colors are ordered by MIC value. This gives a view of changes in the relative abundance of different biocide resistance levels within the total population (note that information on spatial structure is lost in Figure 2c).

In our simulations, biofilm formation happens as follows. First, the initially empty surface acquires a loosely attached layer of microbes, corresponding to a single microhabitat with a population density below the biofilm threshold. Microbes arrive in this layer by immigration, but since the biocide concentration is high close to the surface, most of them rapidly die. Some marginally resistant immigrants are able to replicate, but for our chosen parameter set, even a fully resistant microbe would not initially achieve a population size above the biofilm threshold (see Section 2 and Sinclair et al., 2022). Therefore the loosely attached layer is maintained for some time. During this time, its population fluctuates due to random immigration, proliferation of more resistant microbes and death (Figure 2b). Eventually, one of these population fluctuations pushes the total population size above the biofilm formation threshold N^*^ (Sinclair et al., 2022). At this point, the first microhabitat transitions to the biofilm state and a second microhabitat is added. This triggers the second stage of biofilm development, in which biofilm growth is inevitable. Although the second microhabitat may spend a short time in the loosely attached state^1^ its lower biocide concentration means that it soon transitions to the biofilm state. Subsequent microhabitats are rapidly added, such that the biofilm grows approximately linearly in time.

In the simulation of Figure 2, the first (loosely attached) stage of biofilm formation is characterized by biocide-susceptible micro-organisms (dark colors in Figures 2b,c at early times), but the transition to the second stage (sustained growth) coincides with the arrival of a more biocide-resistant microbe (orange color in Figure 2), which later dominates the biofilm community (Figure 2c). Possibly the immigration of this microbe provided the population fluctuation that triggered the transition to biofilm formation. Furthermore, Figure 2c shows that as the biofilm grows, less resistant microbes also become significant in the community. This suggests a shielding effect: the more resistant microbial type populates the inner parts of the biofilm (Figure 2a), where the biocide concentration is high, allowing for less resistant microbes to contribute to population growth in the outer parts (see the outer layer of darker color in Figures 2a,b).

### 3.2. Diversity of the biofilm community

To further understand changes in community composition during biofilm development (alpha diversity), we investigated the dynamics of three quantitative measures of community structure. The number of species *S* measures how many distinct microbial types (with distinct biocide MIC values) are present in the simulation at any time. The Shannon index $H=-\underset{i}{\sum }p_{i}\ln p_{i}$ measures diversity, taking account of the relative abundances *p*_*i*_ of the species that are present: *H* increases when more species are present, or when their abundances are more evenly distributed. The Shannon equitability *E* = *H*/ln*S* measures the evenness of the distribution of species abundances: a value of 1 means that all species are equally abundant, while a value close to 0 means that one (or a small number of) species is dominant. Figure 3 shows dynamical changes in *S*, *H*, and *E* during biofilm development, averaged over 63 replicate simulation runs.

On average, the number of distinct microbial types *S* within the biofilm community increases in time (Figure 3A). This is consistent with the addition of new microbial types to the community by immigration as the biofilm grows (Figure 2a); since the biocide concentration decreases away from the surface, immigrant microbes are more likely to be viable as the biofilm expands.

However, both the Shannon index *H* and the Shannon equitability *E* decrease, on average, as the biofilm grows (Figures 3B,C). This is consistent with the picture that emerges from Figure 2c, in which the microbial abundance distribution remains highly skewed, even at late times. In other words, the biofilm community is dominated by the most biocide-resistant microbial type, even when it has become thick enough that the biocide concentration at the growing edge is negligible. This is indicative of a priority effect: biocide-resistant organisms that are able to establish early in biofilm development, when the biocide is thin, maintain their dominance at later times even when biocide-resistance is no longer advantageous.

### 3.3. Colonization of the AF surface is stochastic

Repeating our simulations with the same parameter set as in Figure 2, we observed that very different outcomes can arise in replicate simulation runs. Out of 625 replicate simulation runs, 100 (16%) established a biofilm within 6 months' simulated time (defining “biofilm establishment” when the population in the first microhabitat exceeds the biofilm threshold N^*^). Among those simulation runs in which biofilm established, we observed strong variability in the community dynamics. Figure 4 shows the results of three of the replicate simulations in which biofilm established. These simulations vary strongly in the duration of the first, loosely-attached, stage of colonization. Because sustained biofilm growth starts at different times, the final biomass of the biofilm is different in the 3 runs, even though the rate of sustained growth is similar. The 3 replicate runs also show quite different community composition. Replicate A shows a similar pattern to the simulation of Figure 2, in which a somewhat resistant microbe appears around the time of the biofilm transition and later makes up a significant fraction of the community, while coexisting with less resistant micro-organisms. The community of replicate B is far less biocide-resistant. Replicate C, in contrast, contains a highly biocide-resistant organism that almost completely dominates the community, with less resistant microbes being confined to the outer edge of the biofilm. The fact that replicate simulation runs with the same parameter set show qualitatively different outcomes (biofilm vs. no biofilm) as well as different biofilm growth dynamics and community compositions, shows that, in our model, biofouling of the AF surface is a highly stochastic process.

### 3.4. Simulations can predict probability of biofilm establishment on AF surfaces

From an industrial point of view, the waiting time before biofilm establishment on an immersed AF surface is a useful metric for inclusion in tesing and development as well as for establishing in-service paint performance expectations. To probe in more detail the factors influencing biofilm establishment on AF surfaces, we performed 2,000 replicate simulations. For each simulation, we monitored the time of biofilm establishment. Figure 5A shows the fraction of simulations in which biofilm has not yet established, as a function of time (for a parameter set with *c*_max_ = 4.7 ppm). This normalized histogram allows us to obtain the probability distribution *p*_*s*_(*t*) for the time before biofilm establishment (dashed orange line in Figure 5A; this is known in statistical physics as a survival function). Fitting the probability distribution *p*_*s*_(*t*) to an exponential function $p_{s}\left(t\right)=e^{-t/t_{f}}$ allows us to extract the mean biofilm establishment time *t*_*f*_.

Figure 5B shows the *p*_*s*_(*t*) curves for several values of the surface biocide concentration *c*_max_. The corresponding values of the time to biofilm establishment *t*_*f*_ are shown in Figure 5C. The exponential function, shown as the dashed black line, is an excellent fit to the simulation data. As the biocide concentration increases, the exponential function decreases more slowly with time, i.e., the mean time for biofilm establishment increases.

In statistical physics, exponential waiting time distributions like that of Figure 5A are typical of Poisson processes. A Poisson process describes an event whose probability of happening is constant in time. In other words, in our simulations, biofilm can initiate at any time, and the probability of this happening within a given time interval is the same no matter how old the surface is or what its history is. Therefore the timing of biofilm establishment in a particular simulation cannot be predicted; it is controlled by a stochastic process that is history-independent. The exponential waiting time distribution also implies that even if the average time to biofilm establishment is long, there will be some instances of early biofilm formation.

To investigate what factors control the time to biofilm establishment in our simulations, we measured (using thousands of replicate simulations) how the mean biofilm establishment time *t*_f_ depends on the key parameters of our model (Figure 6). As expected, the mean biofilm establishment time increases as the biocide concentration *c*_max_ increases (Figure 6A); this dependence is exponential, suggesting that a small change in biocide concentration can have a large impact on biofilm establishment (note the logarithmic scale on the vertical axes in Figure 6). The biofilm establishment time decreases upon increasing the abundance of biocide-resistant immigrants (Figure 6B) or the immigration rate (Figure 6C); this is consistent with a picture in which the immigration of biocide-resistant organisms plays a key role in the colonization process. It is important to note that the model is not predicting evolution of resistance but selective recruitment and proliferation of higher resistance organisms drawn from the assigned natural distribution. Increasing the rate *r*_det_ at which organisms detach from the outer (loosely attached) edge of the biofilm increases the average biofilm establishment time (Figure 6D), probably because a higher detachment rate makes it harder for the community in the first microhabitat to reach the threshold size for biofilm initiation. Likewise, increasing the biofilm formation threshold, N^*^/*K* (Figure 6E) also increases the biofilm establishment time, simply due to the fact that now more microbes need to replicate/immigrate in order to reach the required density for biofilm to be formed.

Interestingly, the time to biofilm establishment depends non-monotonically on the parameter *r*_max_, which controls both the maximum growth rate for organisms whose MIC is greater than the biocide concentration, and the biocide killing rate for organisms whose MIC is less than the biocide concentration (Figure 6F). This suggests the existence of qualitatively different parameter regimes within the model. Investigation of the community composition within the first microhabitat shows a shift in the distribution of MIC values for low and high *r*_max_ (Supplementary Figure 3). For low values of *r*_max_, there are more sensitive species present (i.e., immigrants with low MIC values, that persist for a while but are eventually killed by the biocide), while for high values of *r*_max_, there are more resistant organisms (i.e., the sensitive immigrants are rapidly killed and only organisms that can grow in this environment survive). In the low *r*_max_, immigrant-dominated, regime, increasing *r*_max_ speeds up the rate at which the biocide-sensitive immigrants are killed, decreasing the population density of the first microhabitat and making it harder for a biocide-resistant immigrant to trigger biofilm formation. In contrast, in the high *r*_max_ regime, increasing *r*_max_ increases the growth rate of the dominant resistant organisms, making biofilm establishment more likely.

## 4. Discussion

### 4.1. Stochastic microbial colonization of an AF surface

Prevention of marine biofouling is a billion-dollar industry. While non-biocidal products exist that provide a high degree of fouling control, for many vessel types, biocidal AF paints continue to be used in the majority of the market (Finnie and Williams, 2010). Microbial biofilm formation is part of the complex marine biofouling challenge, yet few computational models exist for microbial biofilm formation on an AF surface. In this work, we developed, to our knowledge, the first such model, and analyzed its predictions.

The most striking result of our simulations is that colonization of the AF surface can be inherently stochastic, with identical initial conditions producing very different biofilm formation trajectories. In our model, biofilm formation occurs in two stages: initial formation of a loosely-attached layer of microbes, followed by biofilm growth once the population reaches a threshold density. The model biofilm community tends to be dominated by a single more biocide-resistant microbial type, even once the biofilm becomes thick enough that microbes at the growing edge are exposed to a considerably lower biocide concentration—an example of a priority effect. However we also observe in our computer simulations that biocide-resistant microbes shield the community from the biocide, since less biocide-resistant microbes can join the community once it has been established.

For the parameter set used here, a stochastic fluctuation is needed to reach the threshold density for biofilm growth (even for resistant microbes). We find that the waiting times until biofilm establishment follow an exponential distribution, suggesting that biofilm establishment can be modeled as a Poisson process that is inherently unpredictable. In other words, the probability that a biofilm establishes at any time is independent of its history. Investigating the parameter dependence of the average biofilm establishment time, we find that it depends exponentially on the biocide concentration, the immigration rate and the detachment rate. This supports a picture in which immigration of microbes that are sufficiently biocide-resistant to be able to grow in the region close to the surface is a key factor in the triggering of biofilm growth.

For other parameter choices, we would expect our model to behave differently. In particular, if the region close to the surface (the first microhabitat) were able to support a microbial population greater than the threshold density, then the arrival of a resistant microbial type would immediately trigger biofilm growth. In that regime, the biofilm establishment time would simply be controlled by the rate of immigration of sufficiently resistant microbes, and parameters controlling growth behavior close to the surface (e.g., *r*_det_) would not be expected to play a role. We would also expect the average biofilm establishment time to depend linearly on the immigration rate (rather than the exponential dependence seen in our current simulations).

Estimating the accuracy of our model's predictions is difficult, since some of the model parameters are only known within broad ranges (or not at all), and the quantities that we predict (e.g., time to colonization) are rarely measured systematically. We hope that this work will motivate the collection of this kind of data in future, but at present, the aim of our work is primarily to pose the conceptual question of whether, and under what circumstances, microbial colonization of an AF surface could be inherently stochastic.

### 4.2. Biocide concentration profile

In this work, we have assumed, for simplicity, that the biocide concentration decreases exponentially with distance away from the AF paint surface. An exponential profile is consistent with diffusion of the biocide combined with its removal at a fixed rate (perhaps due to chemical degradation in the seawater; see Supplementary material). In reality, however, the concentration profile of biocide around a moving ship coated in AF paint will be determined not only by diffusion and any degradation mechanisms, but also by the fluid flow. The resulting convection-diffusion problem is non-trivial, even if assuming a planar surface with laminar flow in the parallel direction (for example, biocide will accumulate along the flow lines). Including the possibility of turbulent flow would make the model more complicated. There may also be feedback between biofilm growth and the biocide concentration profile, since the biofilm might impede either the release of biocide or its diffusion away from the surface.

For the purpose of the model we have adopted a single biocide gradient profile. We note that most commercial coatings are formulated with two or more biocides, which adds an additional degree of complexity.

### 4.3. Distribution of biocide resistance levels

In this work, we suppose that the MIC values for biocide of microbes in the ocean (immigrants in our model) follow a log-normal distribution. This assumption is based on MIC measurements for bacteria more generally (Turnidge et al., 2006); to our knowledge, little or no investigation has been made of biocide-resistance distributions for marine microorganisms. Furthermore, this distribution might be expected to differ in different geographical regions or in different water bodies (e.g., estuaries compared to open ocean). We also note that biocide-resistance is not the only trait that is relevant to biofilm formation on an AF surface; in future models it might be interesting to include other traits.

More generally, models such as ours are necessarily limited in their representation of biological reality. Here we have characterized microbes only by their biocide resistance ecotype, but in reality, marine biofilms are diverse, containing a mixture of prokaryotes and eukaryotes, where behaviors such as motility, predation, exopolysaccharide production, metabolic interactions and synergy/cooperation may all play a role. A simple model such as that presented here has the virtue of focusing on the effects of differential biocide resistance among marine organisms, but necessarily neglects other possible factors. For this reason, experimental testing of the model predictions would be highly desirable.

### 4.4. Biocide killing

To model microbial growth and biocide killing, we used a pharmacodynamic function proposed by Regoes et al. (2004) to model the response of bacterial populations to antibiotic. This function is convenient because it allows us to characterize microbes by just a single number: the MIC value. All other parameters are assumed to be the same for all microbial types. Moreover, the pharmacodynamic function uses a single parameter (*r*_max_) to describe both the maximal growth rate and the maximal rate of biocide-mediated killing. While this may be true for *Escherichia coli* exposed to cell-wall targeting antibiotics (Lee et al., 2018), it is unlikely to be universally true for marine microbes. In reality, of course, we would expect different marine microbial species to show qualitatively different growth and death dynamics, both in the presence and absence of different biocides. A wide range of bacterial, algal and diatomaceous species have been observed to contribute to marine biofilm formation on modern antifouling paint surfaces (see for example Muthukrishnan et al., 2017; Winfield et al., 2018; Papadatou et al., 2021) and an additional factor is the well-known tolerance of some common fouling species (e.g., *Amphora coffeaeformis*) to some common biocides (e.g., copper-based compounds) (Callow, 1986; Robinson et al., 1992). It would be of interest to measure such growth and killing curves for marine organisms exposed to common biocides and biocide combinations and incorporate this data into computational models.

In our model, the biocide-resistance level might well change when microbes transition from the loosely-attached (“planktonic”) state to the biofilm state of growth (Mah and O'Toole, 2001). For the simulations presented here, this might not change the results significantly, since the biocide mostly plays a role in the first microhabitat, before the transition to the biofilm state. However, it might be an important factor in other parameter regimes. Our model also does not, as yet, include a fitness cost for biocide resistance. This might explain why we see strong priority effects; biocide-resistant organisms that establish early continue to dominate in the later stages of growth, even far from the surface where the biocide concentration is low. It would be interesting to investigate in future how a fitness cost for resistance might alter the predicted species composition.

The fate of dead biomass would also be a relevant factor to consider in future work. Here, we have simply removed dead microbes from the system, implicitly freeing up space (in the form of carrying capacity) for new microbes. Depending on whether the biocide causes lysis, dead microbes might in fact remain within the biofilm, or they might even provide structural elements such as DNA that might strengthen the biofilm. We expect that these factors would have a quantitative, but not a qualitative, effect on our results.

### 4.5. Density-dependent transition to the biofilm state

A major assumption of our model is that the loosely-attached community at the surface transitions to biofilm in a density-dependent manner. Following other modeling work (Moore-Ott et al., 2022; Sinclair et al., 2022), this represents a quorum-sensing mechanism, based on extensive evidence for the involvement of quorum-sensing in biofilm initiation in a variety of microorganisms (Davies et al., 1998; Hammer and Bassler, 2003; Yarwood et al., 2004; Koutsoudis et al., 2006). However, it is also clear that other, non-density-dependent signaling pathways, such as cyclic-di-GMP signaling, are also central in biofilm initiation (Valentini and Filloux, 2016). Moreover, even if quorum-sensing is involved, it is not clear whether a collective transition to biofilm should be triggered by the total microbial density, or whether distinct microbial types might transition when their own densities reach a critical value; in some cases a quorum-sensing transition has even been shown to trigger biofilm formation at low, rather than high, cell density (Hammer and Bassler, 2003; Yarwood et al., 2004). Other factors, such as microbial surface sensing and motility on the surface prior to full attachment *via* the production of expolysaccharide, have also been ignored here (Marshall et al., 1971). The model presented here is clearly a crude approximation, that should be greatly improved as more information emerges on how marine microbes initiate biofilm formation. Nevertheless we hope that our model raises interesting questions that may stimulate further investigation, in particular about the stochastic nature of biofilm initiation.

The parameter *δa* in our model represents the lateral area over which microbes sense the local density and undergo a collective transition to the biofilm state, i.e., the lateral area over which quorum sensing signals operate. A larger value of *δa* would imply a larger carrying capacity and hence a larger value of the biofilm transition population threshold N^*^. In this scenario, stochastic effects would be less important (Sinclair et al., 2022). The spatial range of quorum sensing signals has been addressed in recent work by van Gestel et al. (2021), who concluded that the range depends on the molecular architecture of the quorum-sensing system. A relatively long range (~100*μ*m) is expected for quorum-sensing systems where the signal is not “consumed” upon detection, while a much shorter range is expected for systems where the signal is consumed (van Gestel et al., 2021). In this work, our chosen value for *δa* corresponds to longer-range quorum sensing. In reality, the initiation of a multispecies biofilm might involve a diversity of quorum sensing systems, each one of which might operate over a different spatial range and lead to greater or lesser stochasticity.

### 4.6. Implications for AF paint design

Our simulations raise several interesting questions for the design of AF paint. Firstly, they suggest that microbial biofilm establishment may in some cases be inherently unpredictable, since the underlying processes of immigration of resistant microbes and their transition to the biofilm state, are stochastic. However, our simulations identify key parameters that can increase the average time before biofilm establishment. In particular, for the parameter regime studied here, the biocide concentration is a key factor, upon which the biofilm establishment time depends exponentially. Furthermore, our simulations point to a crucial role for the immigration of biocide-resistant microbes in biofouling. Microbial biofouling on AF paints is globally observed, and recognized species with some biocide resistance (e.g., *Amphora* diatoms) have been recovered from geographically distinct locations. Stochastic microbial fouling processes may be an inherent component of the global challenge for industrial shipping.

## Data availability statement

The raw data supporting the conclusions of this article will be made available by the authors, without undue reservation.

## Author contributions

PS, MC-P, CB, RA, JL, and KR contributed to the study design. RA, MC-P, and CB directed the research. PS performed the research and analyzed the data. RA, MC-P, CB, and PS interpreted the data. PS wrote the manuscript. JL, KR, and AF provided additional guidance on the industrial relevance of the research. All authors edited the manuscript. All authors contributed to the article and approved the submitted version.

## Funding

PS was supported by an EPSRC NPIF studentship, and RA and MC-P were funded by the European Research Council under Consolidator grant 682237 EVOSTRUC. RA acknowledges additional support from the National Biofilms Innovation Centre (BBSRC BB/R012415/1). RA was also supported by the Excellence Cluster Balance of the Microverse (EXC 2051 - Project-ID 390713860) funded by the Deutsche Forschungsgemeinschaft (DFG). CB was funded by the European Research Council under Consolidator Grant 648050 THREEDCELLPHYSICS. For the purpose of open access, the author has applied a Creative Commons Attribution (CC BY) licence to any Author Accepted Manuscript version arising from this submission.

## Conflict of interest

This research was conducted as a collaboration between academic researchers and AkzoNobel, supported by an Engineering and Physical Sciences Research Council National Productivity Investment Fund PhD studentship awarded to PS. However no financial contribution was made by AkzoNobel to the project. L, KR, and AF were employed by International Paint Ltd/AkzoNobel. The remaining authors declare that the research was conducted in the absence of any commercial or financial relationships that could be construed as a potential conflict of interest.

## Publisher's note

All claims expressed in this article are solely those of the authors and do not necessarily represent those of their affiliated organizations, or those of the publisher, the editors and the reviewers. Any product that may be evaluated in this article, or claim that may be made by its manufacturer, is not guaranteed or endorsed by the publisher.

## Footnotes

^1^In some of our simulations the high death rate in the first microhabitat causes net migration of microbes inwards from the second microhabitat, suppressing population growth in the second microhabitat.
