# Supplementary material for: A computational model for microbial colonization of an antifouling surface
Source: Front Microbiol. 2022 Sep 27;13:920014. doi: 10.3389/fmicb.2022.920014 (PMC9551280; doi:10.3389/fmicb.2022.920014)
Supplement: Supplementary file 1 [file Data_Sheet_1.PDF]

Supplementary Material

1 RELATION BETWEEN BIOCIDE MIC VALUE AND KILLING KINETICS

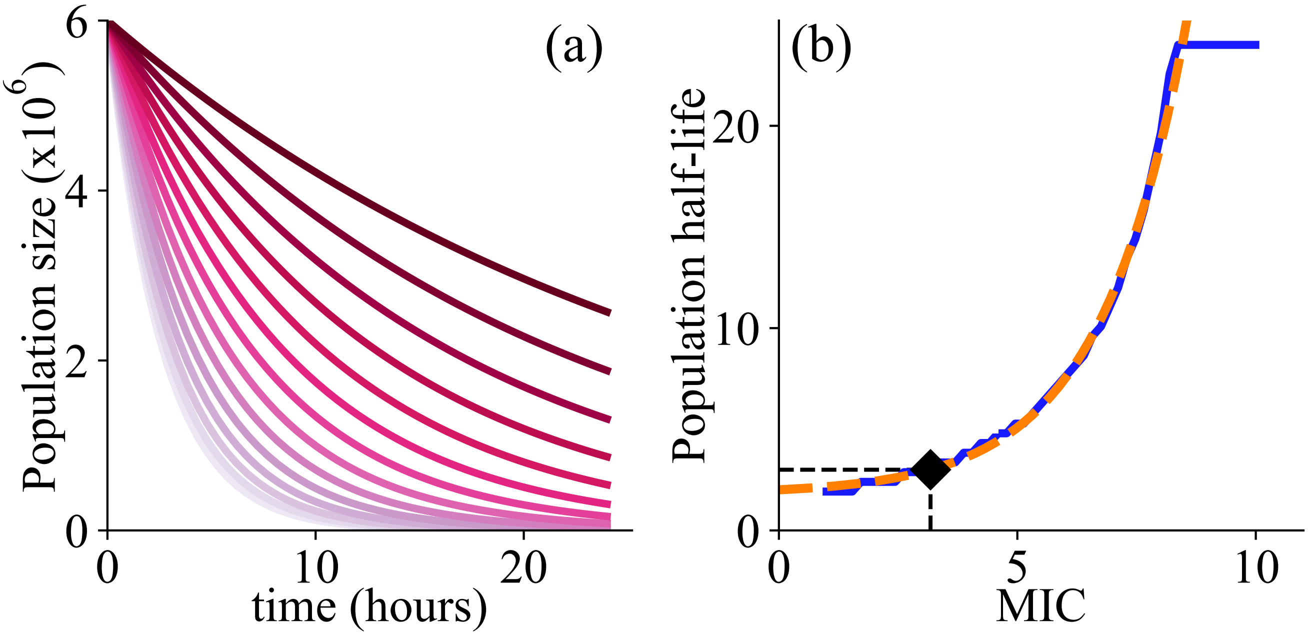

Figure S1. Biocide killing dynamics (a) Simulation of biocide kill curves for a well-mixed population of microbes with uniform MIC value, exposed to 10 ppm biocide. The curves show data for MIC values in the range 1-10 ppm. The darker colours indicate larger MIC values. Only those MIC values which caused the population to halve in size within 24 hours are shown. (b) Population half-life, *i.e.* the time taken for the population size to reduce by half, in hours, is plotted as a function of MIC value, in ppm. The simulated data is shown in blue, and a fit to an exponential function is shown in orange. The dashed lines indicate that we can read off an MIC value compatible with the desired killing kinetics.

To our knowledge, MIC values for marine microbes exposed to biocides are not currently available. However, in some cases experimental data is available on the killing kinetics of marine microbes exposed to biocide (Devilla et al., 2005). Therefore, we performed simulations to relate the biocide MIC values used in our model to biocide killing kinetics. To this end, we simulated a well-mixed population of 600,000 microbes, all with the same MIC value. This population was exposed to biocide at a concentration of 10 ppm. The system was simulated for a time period of 24 hours, and the simulation was repeated for a range of microbial MIC values (1-10 ppm). Fig. S1 (a) shows the resulting killing curves. Fitting these with an exponential function allowed extraction of the half-life, *i.e.* the time for the population size to halve due to biocide killing. Fig. S1 (b) shows the half-life as a function of the MIC (in ppm).

To include microbial diversity in our model, as discussed in the main text, we choose MIC values from a log-normal distribution, given by

$$P(x) = \frac{1}{x\sigma\sqrt{2\pi}} \exp\left(-\frac{(\ln x - \mu)^2}{2\sigma^2}\right).$$

The parameters μ and σ of the log-normal distribution were chosen such that the MIC distribution had a mean value corresponding to the desired average MIC and a specified percentage of the immigrating population had an MIC values greater than $c_{\max}$ (see Table 1 in the main text). In the series of simulations where $c_{\max}$ was varied (Fig. 6(a) in the main text), the parameters μ and σ were kept fixed.

2 BIOCIDE GRADIENT

In our model, we assume that the biocide concentration decreases exponentially with distance from the surface. Such an exponential form of the biocide concentration profile would arise if biocide diffuses away from the surface and is removed at a uniform rate, e.g. by degradation. In such a scenario, the biocide concentration profile would obey the following reaction-diffusion equation

$$\frac{\partial c(z)}{\partial t} = D\frac{\partial^2 c(z)}{\partial z^2} - \zeta c(z)$$

where z is the distance from the surface, $c(z)$ is the local biocide concentration, D is the diffusion constant for biocide in water and ζ is the biocide removal rate. This equation has a steady-state solution of the form $c = Ce^{-\alpha z}$, where $\alpha = \sqrt{\zeta/D}$. This implies that the lengthscale of the biocide concentration profile is controlled by the ratio of the removal rate and the diffusion constant (not by the properties of the paint). The constant C is set by the properties of the paint, since the biocide release rate ξ must match the flux at the surface $-(\partial c/\partial z)_{z=0}$. This implies that $\alpha C = \xi$, hence $C = \xi/\alpha$.

3 IMMIGRATION RATE

Our value for the immigration rate is based upon an experimental study by Fletcher and Loeb (1979) which investigated how the rate of bacterial attachment to a surface was affected by the properties of the surface. Fletcher and Loeb measured the number of bacteria which attached to a surface of area 100 μm^2 that was submerged in seawater with a density of 2.5-5$\times 10^9$ bacteria ml^{-1}, over a period of 2 hours. These measurements were performed for surfaces with various degrees of hydrophobicity, quantified by the contact angle. Assuming that the contact angle of a ship hull is around 80° (Lindholdt et al., 2015), and scaling Fletcher and Loeb's seawater results to correspond to our chosen area of 0.25 mm^2 and a microbial density in the ocean of 10^6 ml^{-1}, we obtain an immigration rate $r_{\rm imm}$ = 20 h^{-1}.

Below, we briefly consider the process of microbial immigration from the ocean to the surface in more detail, to explain why we scale the result of Fletcher and Loeb linearly with the surface area and microbial density.

Let us consider a square patch of surface with side L, area $A = L^2$. We also consider the cube of volume $V = L^3$ immediately above this patch of surface. Supposing that a single microbe is located in the volume V, the time it will take for this microbe to diffuse a distance L out of the volume is given by $\tau = L^2/D$, where D is the diffusion constant of the microbe. If the size of the microbe is a, such that its volume is $V_b = a^3$, the time taken for it to diffuse a distance a out of its own sub-volume is given by $\tau_b = a^2/D$.

Now let us consider splitting the volume V into multiple sub-volumes of size V_b. The number of these sub-volumes is given by $N_s = V/V_b = L^3/a^3$. This results in a grid of $N_s^{\frac{1}{3}} \times N_s^{\frac{1}{3}} \times N_s^{\frac{1}{3}}$ sub-volumes. The number of these sub-volumes which are adjacent to the surface is equal to $N_s^{\frac{2}{3}} = L^2/a^2$.

Within the time τ that it takes for the microbe to leave the volume V, it is able to explore N_e sub-volumes, where $N_e = \tau/\tau_b = (L^2/D) \times (D/a^2) = L^2/a^2$. If the microbe is located in a random sub-volume, the probability that sub-volume is at the surface is

$$p_s = \frac{\text{no. sub-vols at surface}}{\text{total sub-vols}} = \frac{N_s^{\frac{2}{3}}}{N_s}.$$

Therefore, if the microbe explores N_e sub-volumes in time τ, the average number of times it will hit the surface is

$$N_e \times p_s = N_e \frac{N_s^{\frac{2}{3}}}{N_s} = \frac{L^2}{a^2} \frac{L^2/a^2}{L^3/a^3} = \frac{L}{a}.$$

Hence, the rate at which the microbe hits the surface is $L/(\tau a)$. If instead of a single microbe, we have a microbial density of ρ microbes per unit volume, then the volume V will contain ρL^3 microbes. Therefore the overall rate at which microbes hit the surface is $r = \rho L^4/(\tau a)$. Using the following expressions: $\tau = L^2/D$, $D = a^2/(\tau_b)$, $L^2 = A$, the immigration rate can be written as $r_{\text{imm}} = \rho A a/\tau_b$. This expression scales linearly with ρ, A and a, as expected.

4 DETACHMENT RATE AND THRESHOLD FOR BIOFILM FORMATION

In our model, we include a ‘detachment’ process, in which microbes are lost from the outermost microhabitat. This aims to account for processes such as sloughing or grazing which might occur in marine biofilms, as well as microbial detachment via diffusive motion. A broad range of detachment rates (between 10^0 and 10^{-4} hour^{-1}) have been reported in the literature (Kiørboe et al., 2003; Boe-Hansen et al., 2002), making it difficult to set this parameter based on literature values.

We also include in the model a threshold population size N^*, above which the transition to biofilm is triggered. This is a hypothetical model construction, based on previous theoretical work (Sinclair et al., 2022). Therefore the value of N^* cannot be obtained from experimental literature.

To deal with these parameter uncertainties, we took a phenomenological approach to choosing both the detachment rate and the threshold density. The growth of biofilm in the absence of biocide was simulated for a range of values of the detachment rate r_{det} and the biofilm formation threshold N^* (setting the other parameters as in Table 1 of the main text). In these simulations, we measured the biofilm thickness after a simulated time of 10 days (Fig.S2). These values were compared to the experimental measurements of Dobretsov and Thomason (2011) for the growth of marine biofilms. Based on this we chose values of $N^* = 0.75 \times K$ and $r_{\text{det}} = 0.22 \times g_{\text{max}}$.

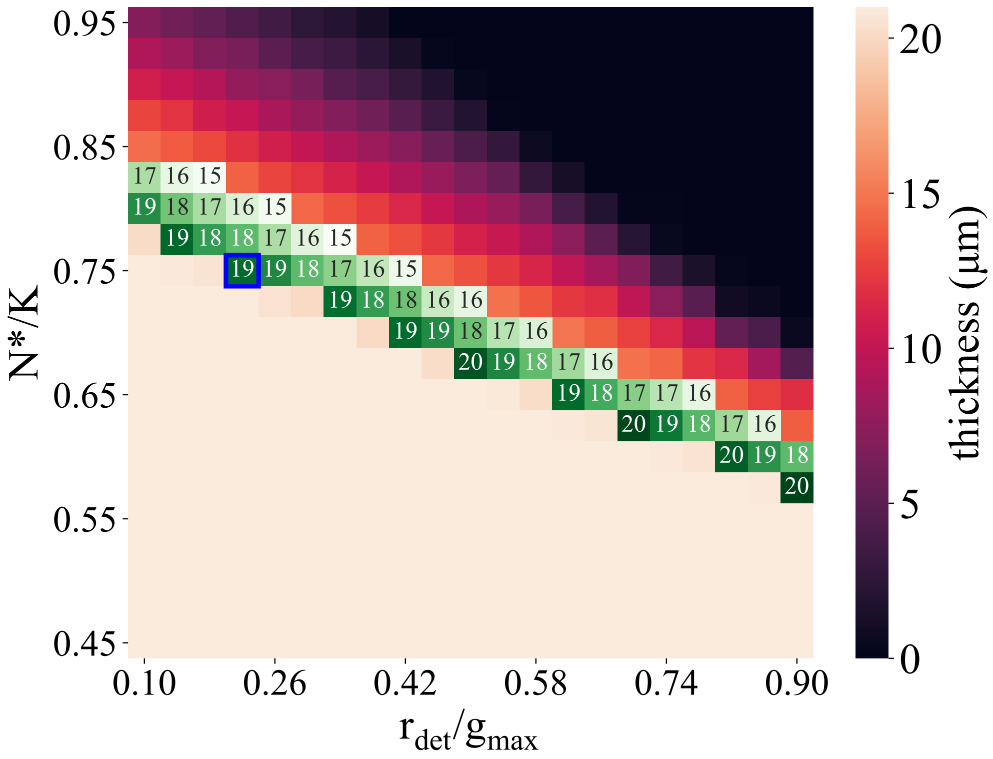

Figure S2. Parameter dependence of biofilm growth in the absence of biocide The heatmap shows the simulated biofilm thickness in (μm) after 10 days, as a function of N^*/K and $r_{\rm det}/g_{\rm max}$ for biofilms growing on a biocide-free surface. N^* and $r_{\rm det}$ were varied; other parameters are as in Table 1 of the main text. The green region corresponds to parameter pairs which gave thicknesses in agreement with the experimental data of Dobretsov and Thomason (2011); with the numbers indicating the simulated thickness in μm. The parameter pair used in the simulations presented in the main text is highlighted in blue : $N^*/K = 0.75$, $r_{\rm det}/g_{\rm max} = 0.22$.

5 EFFECT OF PROLIFERATION/KILLING RATE PARAMETER ON COMMUNITY COMPOSITION

In our model, the parameter $r_{\rm max}$ controls both growth rate (if the biocide concentration is below the MIC) and death rate (if the biocide concentration is above the MIC). Fig. S3 shows that for the lower value of $r_{\rm max}$ the community contains more microbes with low MIC values, which are eventually killed by the biocide. However, for the higher value of $r_{\rm max}$, these sensitive immigrants are rapidly killed and instead the community contains more resistant species. This change in community composition can explain the non-monotonicity in the dependence of time to colonization on $r_{\rm max}$ exhibited in Fig. 6(f) of the main text.

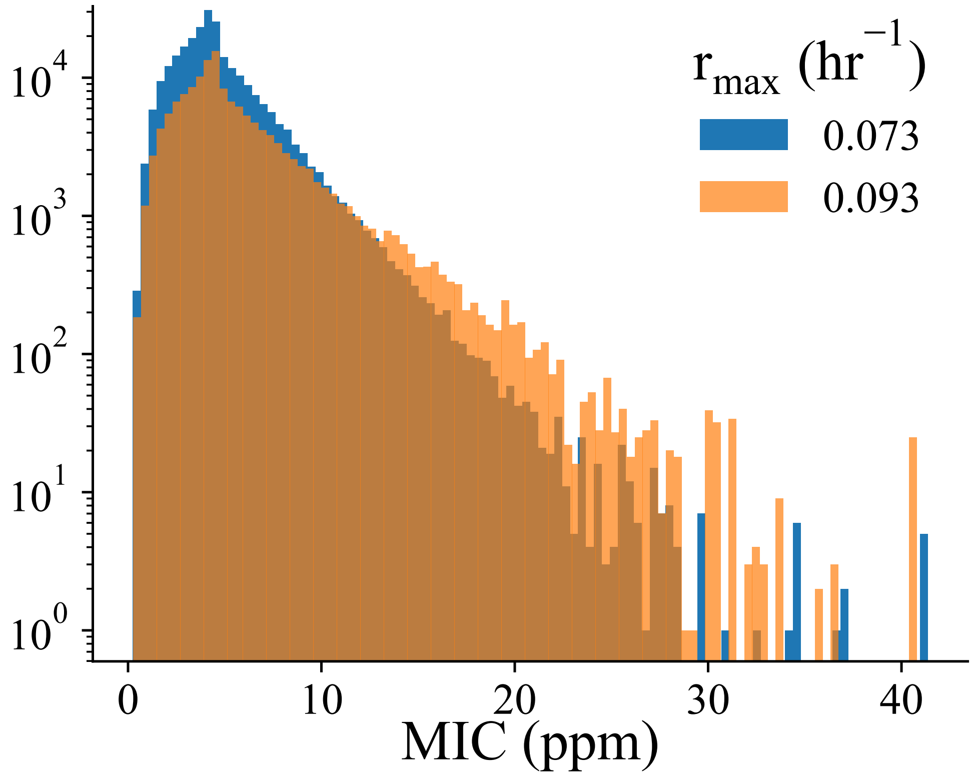

Figure S3. Distribution of MIC values in the first microhabitat, for high and low values of the parameter $r_{\rm max}$. Simulations were run for $r_{\rm max} = 0.073$ hr^{-1} (blue data) and 0.093 hr^{-1} (orange data). All other parameters were as in Fig. 6(f) of the main text. The simulations were terminated when the first microhabitat reached the biofilm threshold. Histograms are plotted of the MIC values for the community in the first microhabitat, at the end of these simulations. Note the log scale on the vertical axis.

6 SIMULATION ALGORITHM

Our simulations use a version of Gillespie's τ-leaping algorithm (Gillespie, 2001), modified to avoid negative population sizes (Cao et al., 2005). Although this is a standard algorithm, for clarity, we set it out in detail here in the context of our model. The algorithm proceeds as follows:

1. An initial value for the timestep τ is selected. In this model, $\tau = 0.2$ hours is used.
2. Iterating over each microbe j in each microhabitat i, the algorithm predicts the number of replications or deaths ($b_{i,j}$ and $d_{i,j}$ respectively) that will be experienced by that microbe in time τ. These numbers are determined by sampling from a Poisson distribution with mean $r_{i,j} \times \tau$, where $r_{i,j}$ is the replication rate or the death rate of microbe j in microhabitat i.
3. In order to avoid negative populations (a microbe cannot die twice), if step 2 results in any predictions of more than one death or replication (*i.e.* any values of $b_{i,j}$ and $d_{i,j} > 1$), then the algorithm returns to step 1 and repeats the process, with a smaller timestep $\tau \to \tau/2$. This also happens if step 2 predicts that a microbe will both replicate and die in the same timestep.
4. Iterating again over each microbe j in each microhabitat i, the algorithm now determines which microbes will migrate between adjacent microhabitats during the timestep τ. To this end, each microbe is allocated a 'migration number' $m_{i,j}$ obtained by sampling from a Poisson distribution with mean

$r_{\mathrm{mig}} \times \tau$. As microbes in the inner or outer edge microhabitats can only migrate in one direction, their $m_{i,j}$ values are instead allocated by sampling from a Poisson distribution with mean $0.5 \times r_{\mathrm{mig}} \times \tau$.

5. Next, the algorithm iterates over microbes j in the outermost microhabitat, determining which microbes will detach in time τ. These microbes are allocated a ‘detachment number’ z_j which is determined by sampling from a Poisson distribution with mean $r_{\mathrm{det}} \times \tau$.
6. To avoid the possibility of a microbe undergoing more than one migration or detachment event in the same timestep, the migration and detachment allocations are handled in a similar way to step 3; if a microbe is designated to migrate or detach more than once ($m_{i,j} > 1$ or $z_j > 1$), the timestep is halved, $\tau \to \tau/2$, and the algorithm returns to step 2.
7. The events that have been determined in steps 2, 4 and 5 are then carried out. Thus, microbe j in microhabitat i either replicates $b_{i,j}$ times (1 or 0), or dies $d_{i,j}$ (1 or 0) times. If the microbe is in the edge microhabitat then it will detach if $z_j = 1$. For migration, if $m_{i,j} > 0$ then microbe j in microhabitat i is sent to either microhabitat $i + 1$ or $i - 1$ with equal probability, unless the microbe is in an edge microhabitat, in which case it can only migrate in one direction. Dead or detached microbes are removed from the system. Microbes that are designated to die or detach in that timestep will only carry out that event and no other events. Microbes that are designated to both replicate and migrate in the same timestep will first replicate, then the mother cell will migrate.
8. The number of microbes that immigrate into the outermost microhabitat during timestep τ is then sampled from a Poisson distribution with mean $r_{\mathrm{imm}} \times \tau$. The MIC values of these microbes are assigned by random sampling from the lognormal distribution described in section 1 above and in the main text.
9. If the number of microbes in the outermost microhabitat has reached the threshold for biofilm formation (*i.e.* it is $\geq N^*$), then another microhabitat is added to the system, in adjacent to the current outermost microhabitat. This new microhabitat is allocated as the new outermost microhabitat and is the one which microbes will immigrate into, and detach from, in the next timestep.
10. The time elapsed in the simulation is updated to $t \to t + \tau$. For the next timestep, τ returns to its original value of 0.2 hours.

REFERENCES

Boe-Hansen, R., Albrechtsen, H.-J., Arvin, E., and Jørgensen, C. (2002). Bulk water phase and biofilm growth in drinking water at low nutrient conditions. *Water Res.* 36, 4477 – 4486

Cao, Y., Gillespie, D. T., and Petzold, L. R. (2005). Avoiding negative populations in explicit Poisson tau-leaping. *J. Chem. Phys.* 123, 054104

Devilla, R. A., Brown, M. T., Donkin, M., Tarran, G. A., Aiken, J., and Readman, J. W. (2005). Impact of antifouling booster biocides on single microalgal species and on a natural marine phytoplankton community. *Mar. Ecol. Prog. Ser.* 286, 1–12

Dobretsov, S. and Thomason, J. C. (2011). The development of marine biofilms on two commercial non-biocidal coatings: a comparison between silicone and fluoropolymer technologies. *Biofouling* 27, 869–880

Fletcher, M. and Loeb, G. (1979). Influence of substratum characteristics on the attachment of a marine pseudomonad to solid surfaces. *Appl. Environ. Microbiol.* 37, 67–72

Gillespie, D. T. (2001). Approximate accelerated stochastic simulation of chemically reacting systems. *J. Chem. Phys.* 115, 1716–1733

Kiørboe, T., Tang, K., Grossart, H.-P., and Ploug, H. (2003). Dynamics of microbial communities on marine snow aggregates: Colonization, growth, detachment, and grazing mortality of attached bacteria. *Appl. Environ. Microbiol.* 69, 3036–3047

Lindholdt, A., Dam-Johansen, K., Yebra, D., Olsen, S., and Kiil, S. (2015). Estimation of long-term drag performance of fouling control coatings using an ocean-placed raft with multiple dynamic rotors. *J. Coatings Tech. Res.* 12, 975–995

Sinclair, P., Brackley, C. A., Carballo-Pacheco, M., and Allen, R. J. (2022). A model for quorum-sensing mediated stochastic biofilm formation. *bioRxiv* , 10.1101/2022.03.23.485488
